# Spontaneous unilateral quadruplet tubal ectopic pregnancy

**DOI:** 10.4274/tjod.galenos.2020.64624

**Published:** 2020-07-29

**Authors:** Burak Karadağ, Burcu Aykan Yüksel, Cemil Gürses, Selim Karataş

**Affiliations:** 1 University of Health Sciences Turkey, Antalya Training and Research Hospital, Clinic of Obstetrics and Gynecology, Antalya, Turkey; 2 University of Health Sciences Turkey, Antalya Training and Research Hospital, Clinic of Radiology, Antalya, Turkey

**Keywords:** Ectopic pregnancy, multiple gestations, quadruplet

## Abstract

Ectopic pregnancy (EP) is defined as the implantation of the fertilized ovum outside the uterine cavity. Importantly, the implantation site is tubal in 95% of the cases. Multiple EPs are extremely rare. We present a case of a 25-year-old patient, gravida 2 para 1, with amenorrhea accompanied by the complaints of vaginal bleeding and abdominal pain. She was admitted to the emergency department. Trans-vaginal ultrasound revealed a left ovarian anechoic cyst of 30 mm and four embryos in the right tube with positive cardiac activities. An emergency laparotomy found the rupture of tubal pregnancy on the right side, which ultimately led to hemo-peritoneum. Therefore, we performed right salpingectomy. This is the first well-documented case of a patient with spontaneous unilateral quadruplet tubal EP.

## Introduction

Ectopic pregnancy (EP) occurs when developing blastocysts are implanted outside the endometrium in the uterine cavity^(1)^. The primary site of implantation is the fallopian tube, generally in the ampullary region, in more than 98% cases of EPs. The other cases of EPs occur in the abdominal cavity, on the ovary, or in the cervix^(2,3)^. EP occurs in 1%-2% of all the pregnancies^(4)^. Interestingly, 6%-16% of the women admitted to the emergency department with first-trimester bleeding, pain, or both are diagnosed with EP^(2,5,6)^. A history of EP, tubal surgery, tubal ligation, tubal pathology, in utero Diethylstilbestrol exposure, or the current use of intrauterine device (at the time of admission) are the prominent risk factors of EP^(6)^. The main complication of EP is tubal rupture, which can eventually cause massive intra-abdominal bleeding and possibly death^(1,2)^.

Multiple EPs are rare and might result from simultaneous bilateral ovulation or superfetation, with or without trans-peritoneal migration^(7)^. Unilateral and bilateral twin ectopic gestations have been reported in the literature. Additionally, up-to-date cases and several spontaneous unilateral triplet tubal pregnancies have also been documented^(7,8)^.

To our knowledge, this is the first well-documented case of a patient with spontaneous unilateral quadruplet tubal EP.

## Case Report

**Figure 1:**
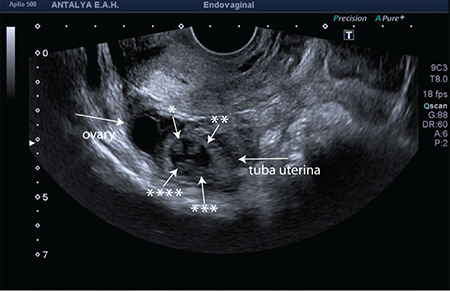
Sonographic appearance of right tubal quadruplet ectopic pregnancy. *indicates the order number of embryos in the tubal gestational sac

**Figure 2:**
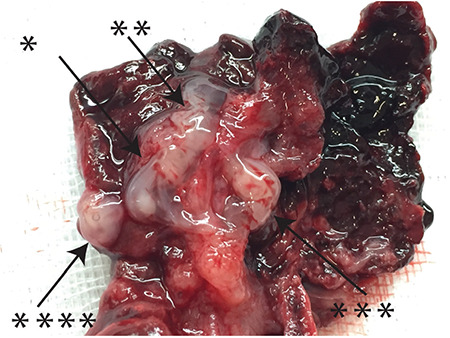
Postoperative gross examination of the tubal mass. *indicates the order number of embryos in the postoperative specimen

A 25-year-old patient, gravida 2 para 1, was admitted to the emergency department at seven weeks plus two days of amenorrhea accompanied with the complaints of vaginal bleeding and abdominal pain. She did not carry any of the risks for EP and was not using any method for contraception. Physical examination revealed that her blood pressure was 80/50 mmHg with a pulse rate of 110 beats per minute. It also reported postural hypotension. There was also the presence of abdominal guarding and rebound tenderness. Vaginal examination revealed slight-moderate bleeding; however, she felt pain mainly on the right side during the bimanual examination. Trans-vaginal ultrasound revealed left ovarian anechoic cyst of 30 mm and four embryos in the right tube (Figure 1). All the embryos had cardiac activities and crown rump length measurements were consistent with average seven weeks of pregnancy. There was free fluid and coagulum in the pouch of Douglas and the right para-ovarian space. Hemoglobin and hematocrit levels were 9.8 gr/dL and 29%, respectively. Clinical and laboratory findings were consistent with the tubal rupture. More importantly, the presence of hemodynamic instability led us to perform an emergency laparotomy. Informed consent was obtained from the patient. Under general anesthesia, the abdominal cavity was accessed via Pfannenstiel incision. Further exploration revealed that tubal pregnancy on the right side had ruptured, which ultimately led to hemo-peritoneum with approximately 800 mL of blood and coagulum. The left tube and the cornual portions of the uterus were intact. Right salpingectomy was performed, and all the materials were sent to pathology. The final pathology report revealed tubal quadruplet pregnancy and chronic salpingitis. A detailed gross examination of the right tube revealed that its diameter was 40 mm in its widest part and four embryos were identified between the pieces of coagulum (Figure 2).

## Discussion

Since the publishing of Dr. Wilmer Krusen’s report of a patient with spontaneous unilateral triplet tubal pregnancy in 1902, several reports of similar triplet ectopic pregnancies have also been published^(9)^. Multiple ectopic pregnancies can also present as a component of a heterotopic pregnancy, which can be more difficult to diagnose. The prevalence of HP has increased from 1 in 30,000 normal gestations to 152 in 100,000, which is believed to be related to the advent and growing use of technologies such as ovulation induction and assisted reproductive technology^(10)^. Other important risk factors for EP include a history of EP, damage to the fallopian tubes, pelvic infection, pelvic or fallopian tube surgery, and infertility. Other less significant risk factors include cigarette smoking and patients older than 35 years^(11)^. But one half of all the women who were diagnosed with EP do not present any known risk factors like the presented case^(12)^.

The management of an EP depends on the hemodynamic status of the patient, the location, gestational age, the activity of the trophoblast human chorionic gonadotropin-beta, as well as the presence of a concomitant pregnancy (heterotopic pregnancy) and obstetric history of the patient. Methotrexate treatment can be considered for women with a confirmed diagnosis of EP who are hemodynamically stable, who have an unruptured mass, and who do not have absolute contraindications for methotrexate^(11)^. The decision for the surgical management or medical management of EP should be guided by the initial clinical, laboratory, and radiologic data as well as patient-informed choice based on a discussion of the benefits and risks of each approach. Also, women who are treated with methotrexate therapy should be counseled about the importance of follow-up. In our case, we chose to perform salpingectomy through laparotomy based on the volume of the hemo-peritoneum, the patient’s hemodynamic instability, ultrasound-documented viable four ectopic pregnancies, and signs of tubal rupture.

Our case is the first well-documented report of a patient with spontaneous live quadruplet tubal EP, and this case report highlights that multiple ectopic pregnancies can occur in relation to assisted reproductive technology, but they can also occur spontaneously.
